# Further Elucidation of Galactose Utilization in *Lactococcus lactis* MG1363

**DOI:** 10.3389/fmicb.2018.01803

**Published:** 2018-08-03

**Authors:** Ana Solopova, Herwig Bachmann, Bas Teusink, Jan Kok, Oscar P. Kuipers

**Affiliations:** ^1^Department of Molecular Genetics, Groningen Biomolecular Sciences and Biotechnology Institute, University of Groningen, Groningen, Netherlands; ^2^Faculty of Earth and Life Sciences, Systems Bioinformatics IBIVU/NISB, Vrije Universiteit Amsterdam, Amsterdam, Netherlands

**Keywords:** galactose transporter, PTS, PEP-transferase system, *Lactococcus lactis*, GalP

## Abstract

Since the 1970s, galactose metabolism in *Lactococcus lactis* has been in debate. Different studies led to diverse outcomes making it difficult to conclude whether galactose uptake was PEP- or ATP- dependent and decide what the exact connection was between galactose and lactose uptake and metabolism. It was shown that some *Lactococcus* strains possess two galactose-specific systems – a permease and a PTS, even if they lack the lactose utilization plasmid, proving that a lactose-independent PTS^Gal^ exists. However, the PTS^Gal^ transporter was never identified. Here, with the help of transcriptome analyses and genetic knock-out mutants, we reveal the identities of two low-affinity galactose PTSs. A novel plant-niche-related PTS component Llmg_0963 forming a hybrid transporter Llmg_0963PtcBA and a glucose/mannose-specific PTS are shown to be involved in galactose transport in *L. lactis* MG1363.

## Introduction

The conversion of the disaccharide lactose into lactic acid is the main function of lactic acid bacteria during industrial fermentations. The first step of lactose catabolism is the hydrolysis of the molecule resulting in production of glucose and galactose or galactose-phosphate. While glucose readily enters glycolysis, further metabolism of galactose can be less efficient in some strains. As a result, the galactose moiety might even be excreted to the growth medium and used up later (Benthin et al., 1994; Pool et al., 2006). Lactic acid bacteria can import and utilize galactose in two main ways. When it is taken up by a permease, it is channeled through the Leloir pathway reactions. The high-affinity galactose permease GalP and the enzymes of the Leloir pathway are encoded in one gene cluster in *L. lactis*, with a gene order that reflects exactly the order of biochemical reactions of galactose degradation (Grossiord et al., 1998). Imported galactose is modified by galactose mutarotase (encoded by *galM*) into α-D-galactose as only this galactose anomer can serve as a substrate for galactokinase (*galK*), which catalyzes the phosphorylation of internalized galactose. Galactose-1-P uridyltransferase (*galT*) converts galactose-1-P into UDP-galactose using UDP-glucose as a substrate. UDP-glucose 4-epimerase (*galE*) catalyzes the interconversion between UDP-galactose and UDP-glucose. Glucose-1-P is then converted into glucose-6-P by phosphoglucomutase and enters glycolysis.

The second way to import galactose is via a PEP-transferase system (PTS). Here, two options exist: one is to use the lactose-specific PTS^LacEF^ or, alternatively, to employ a galactose-specific PTS. Both routes usually feed into the tagatose-6-P pathway, which starts with conversion of galactose-6-P to tagatose-6-P by galactose-6-P isomerase (*lacAB*), then continues with phosphorylation of the compound into tagatose-1,6-BP by tagatose-6-P kinase (*lacC*), and finally ends with cleavage of the latter into trioses by tagatose-1,6-BP aldolase (*lacD*) (Grossiord et al., 1998; Zeng et al., 2010). Sometimes the two pathways are intertwined. In *S. mutans*, which possesses the Leloir and lactose PTS-Tag-6-P pathways, the two seem to interact and both are necessary for growth of this bacterium in galactose-containing medium (Abranches et al., 2004). The Leloir pathway reactions in *S. mutans* appear to be coupled to the transport of galactose via two PTSs: mannose-specific-PTS (encoded by *manLMN*) and lactose-specific PTS (*lacFE*). It has been reported that the Tag-6-P pathway alone is not sufficient for growth of this bacterium on galactose; additionally, the Leloir pathway should be activated, presumably because of the role of the intermediate product UDP-galactose in cell envelope maintenance (Zeng et al., 2010).

*Lactococcus lactis* MG1363 and its direct derivative NZ9000 do not possess a PTS^LacEF^ or the Tag-6-P pathway. A permease GalP is the main galactose uptake system; when *galP* is deleted from the chromosome, a yet unidentified PTS system remains active. After galactose internalization by the PTS and consequent phosphorylation of the molecule to galactose-6-phosphate, it is dephosphorylated by an unidentified hexose-P phosphatase. The dephosphorylation step makes this route energetically inefficient, but it is a prerequisite for entering the Leloir pathway reactions, the only way to metabolize galactose in *L. lactis* MG1363 (Neves et al., 2010). The PTS can only import galactose when the concentration of this sugar in the environment is high, as it exhibits a lower affinity for galactose than GalP (Grossiord et al., 2003; Neves et al., 2010). Although, the presence of a galactose-importing phosphotransferase activity in *L. lactis* has been convincingly shown in previous studies, the transporter itself was never identified (LeBlanc et al., 1979; Thompson, 1980; Neves et al., 2010).

*Lactococcus lactis* MG1363 possesses 12 operons encoding various PTS components that could potentially import galactose (Wegmann et al., 2007). Some of these clusters contain genes putatively involved in plant sugar uptake and utilization reactions and they seem to have decayed during adaptation of *L. lactis* to a milk environment. However, it is known that such cryptic gene clusters can be revived and gain new functions when a certain selection pressure is applied (Solopova et al., 2012). The aim of this work was to study PTSs with unassigned specificities and to finally identify the missing galactose transporter in *L. lactis* MG1363.

## Materials and Methods

### Microbial Strains and Growth Conditions Used

*Lactococcus lactis* MG1363 (Gasson, 1983) derivatives were grown as standing cultures at 30°C in M17 broth (Difco™, Sparks, MD, United States) or in chemically defined medium (CDM PC) (Goel et al., 2012) supplemented with 5, 25, or 50 mM glucose, fructose, or galactose. GM17-agar plates were prepared by adding 1.5% (wt/vol) agar to M17 with 25 mM glucose. When appropriate, erythromycin (Sigma-Aldrich, St. Louis, MO, United States) was used at 1 μg ml^-1^. *E. coli* DH5α was used as a cloning host and was grown in LB medium at 37°C or on LB medium solidified with 1.5% (wt/vol) agar. For plasmid selection, 150 μg ml^-1^ erythromycin (Sigma) was added. Strains and plasmids used in this study are listed in **Table 1**.

**Table 1:** Strains and plasmids used in this study.

Strain or plasmid	Description	Source or reference
*E. coli* DH5α	F^-^ 80*lacZ*ΔM15 Δ(*lacZYA-argF*) U169 *rec1A end1A hsdR17 gyrA96 supE4 4thi-1 relA1*	Invitrogen (Carlsbad, CA, United States)
***L. lactis* strains**		
MG1363	*L. lactis* subsp. *cremoris*; plasmid-free derivative of NCDO712	Gasson, 1983
HB70 ( = MGGal+)	Chemically mutagenized MG1363 derivative carrying 29 mutations in the genome	This study
MGΔ*galP*	MG1363 carrying a chromosomal deletion of *galP*	This study
MGΔ*galP*Δ*ptcC*	MG1363 carrying a chromosomal deletion of *galP* and *ptcC*	This study
MGΔ*galP*Δ*ptcBA*	MG1363 carrying a chromosomal deletion of *galP* and *ptcBA*	This study
MGΔ*galP*Δ*llmg_0963*	MG1363 carrying a chromosomal deletion of *galP* and *llmg_0963*	This study
MGΔ*galP*Δ*llmg_0963*Δ*ptcBA*	MG1363 carrying a chromosomal deletion of *galP, ptcBA*, and *llmg_0963*	This study
MGΔ*galP*Δ*llmg_0963*Δ*ptnCD*	MG1363 carrying a chromosomal deletion of *galP, llmg_0963*, and *ptnCD*	This study
MGΔ*galP*Δ*llmg_0963*Δ*ptcBA*Δ*ptnCD*	MG1363 carrying a chromosomal deletion of *galP, llmg_0963, ptcBA*, and *ptnCD*	This study
MGΔ*galP*Δ*ptcBA*Δ*ptnCD*	MG1363 carrying a chromosomal deletion of *galP, ptcBA*, and *ptnCD*	This study
**Plasmids**		
pCS1966	Integration vector for *L. lactis*; erythromycin resistance	Solem et al., 2008
pCS1966-*galP′*	pCS1966 containing *galP* deletion construct	This study
pCS1966-*ptcBA′*	pCS1966 containing *ptcBA* deletion construct	This study
pCS1966-*ptcC′*	pCS1966 containing *ptcC* deletion construct	This study
pCS1966-*ptnCD′*	pCS1966 containing *ptnCD* deletion construct	This study
pCS1966-*0963′*	pCS1966 containing *llmg_0963* deletion construct	This study

### General DNA Techniques

DNA manipulations were done essentially as described (Sambrook et al., 1989). Plasmid DNA and PCR products were isolated and purified using the High Pure Plasmid Isolation Kit (Roche Applied Science, Mannheim, Germany) according to the manufacturer’s instructions.

Restriction enzymes, T4 DNA ligase and Taq DNA-polymerase were obtained from Thermo Fisher Scientific Baltics (Vilnius, Lithuania) and used according to the supplier’s instructions. Phusion DNA Polymerase was purchased from New England Biolabs (Ipswich, United Kingdom). PCR were performed in a Biorad thermal cycler (Hercules, CA, United States) using *L. lactis* MG1363 chromosomal DNA as the template, unless described otherwise.

### Isolation and Characterization of MGGal^+^

*Lactococcus lactis* MG1363 was chemically mutagenized using 25 mM ethyl methanesulfonate (EMS) and then propagated in emulsion at 30°C as described earlier (Bachmann et al., 2013) with the difference that the CDM medium was supplemented with 5 mM galactose instead of glucose. After 22 propagation cycles individual colonies were isolated. MGGal^+^ derived from a single colony and was selected for further analysis as it showed a decreased growth rate but a significant increase in the final OD_600_ in CDM-gal. Full genome re-sequencing and analysis and metabolite analysis were carried out as described earlier (Bachmann et al., 2013).

### Construction of *L. lactis* Deletion Strains

Primer sequences used in this study are shown in Supplementary Table 1. The PCR products obtained with primer pairs KogalP1F/KogalP2R and KogalP3F/KogalP4R were cloned together as *Xba*I/*Bam*HI and *Bam*HI/*Xho*I restriction fragments in *Xba*I/*Xho*I-restricted integration vector pCS1966 (Solem et al., 2008) resulting in pCS1966-*galP′*. PCR products obtained with primers KoPtcBA1F/KoPtcBA2R and KoPtcBA3F/KoPtcBA4R were cloned as *Xba*I/*Bam*HI and *Bam*HI/*Xho*I restriction fragments in *Xba*I/*Xho*I-restricted pCS1966, resulting in pCS1966-*ptcBA′*. KoptcC1F/KoptcCS2Rev and KoptcC3F/KoptcC4Rev PCR products were cloned as *Xba*I/*Bam*HI and *Bam*HI/*Xho*I restriction fragments in *Xba*I/*Xho*I-restricted pCS1966, resulting in pCS1966-*ptcC′*. The PCR products obtained with primer pairs KoptnCD1F/KoptnCD2R and KoptnCD3F/KoptnCD4R were cloned as *Xba*I/*Bam*HI and *Bam*HI/*Xho*I restriction fragments in *Xba*I/*Xho*I-restricted pCS1966, resulting in pCS1966-*ptnCD′*. The flanking regions of *llmg_0963* were amplified using Ko09631F/Ko09632R and Ko09633F/Ko09634R. These fragments were ligated into pCS1966 via *Xba*I/*Bam*HI and *Bam*HI/*Kpn*I restriction sites. The resulting vector was designated pCS1966-*0963*′. All the pCS1966 derivatives were obtained and maintained in *E. coli* DH5α.

Vectors pCS1966-*galP′*, pCS1966-*ptcBA′*, pCS1966-*ptcC′*, pCS1966-*ptnCD′*, or pCS1966-*0963′* were introduced in *L. lactis* MG1363 and its derivatives via electroporation (Holo and Nes, 1995); a two-step homologous recombination event was induced by growing cells on selective SA medium plates (Jensen and Hammer, 1993) supplemented with 20 μg ml^-1^ 5-fluoroorotic acid hydrate (Sigma) (Solem et al., 2008). The chromosomal structure of all deletion strains was confirmed by PCR analysis and sequencing of the modified regions.

### Transcriptome Analyses

Transcriptome analysis was performed using full-genome *L. lactis* MG1363 DNA-microarrays as described previously (Kuipers et al., 2002). MGGal^+^ cells were grown in CDM supplemented with either 5 mM galactose or 5 mM fructose, and harvested at two time points [beginning of the exponential phase, at an optical density at 600 nm (OD_600_) of 0.1 and late-exponential phase, at OD_600_ of 0.35]. RNA from cells growing on galactose was compared to RNA from cells of the same growth phase on fructose. DNA-microarray slides were scanned with a Genepix 4,200 laser scanner at 10 μm resolution. Slide images were analyzed using ArrayPro 4.5 (Media Cybernetics, Inc., Silver Spring, MD, United States). Processing and normalization (LOWESS spotpin-based) of slides was done with the *MicroPrep* software (van Hijum et al., 2003). Differential expression tests were performed on expression ratios with a local copy of the Cyber-T implementation of a variant of the *t*-test. A gene was considered differentially expressed when the Bayesian *P*-value < 0.001.

### Growth Experiments

To follow the growth of mutant strains, overnight cultures were diluted 40-fold and grown as 0.2 mL cultures in CDM supplemented with galactose in 96-well microtiter plates at 30°C and monitored with an Infinite 200 PRO 16 microplate spectrophotometer (Tecan Group, Ltd., Männedorf, Switzerland). Growth was monitored by measuring the OD_600_.

## Results

### Isolation of MGGal^+^

**FIGURE 1:**
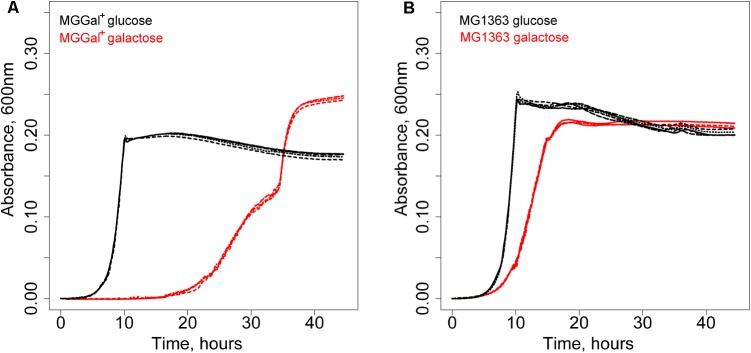
Growth of *Lactococcus lactis* strains in CDM supplemented with 5 mM of galactose (red) or glucose (black). **(A)** *L. lactis* MGGal^+^. **(B)** *L. lactis* MG1363.

A derivative of *L. lactis* MG1363 was isolated using chemical mutagenesis and a high biomass-yield selection protocol (see section “Materials and Methods”; Bachmann et al., 2013). This method eliminates the competition between individual cells through compartmentalization of single cells in water-in-oil emulsion droplets. It allows selection of mutants with an increased number of offspring rather than increased growth rate (Bachmann et al., 2013). The isolated strain grew slower but was able to reach a higher optical density at 600 nm on galactose than its parent strain. Curiously, this strain, designated MGGal^+^, showed a biphasic growth pattern in CDM, supplemented with 5 mM galactose (CDM-gal) (**Figure 1**). The first growth phase starts after a prolonged lag-phase (around 20 h) and is characterized by a higher growth rate (μ = 0.25). After an OD of 0.15 is reached, the growth slows down and the second growth phase (μ = 0.15) follows. As estimated by Neves et al. (2010) the transporters involved in galactose uptake in *L. lactis* NZ9000 (a derivative of MG1363) have Km values in the mM range (∼6 mM) for an unidentified gal-PTS and in μM range for GalP. We hypothesized that the two distinct growth phases could be a result of the activity of two galactose transporters with different affinities for galactose, i.e., when the galactose concentration is high, the low-affinity and the high-affinity transporters are functional; when the concentration of this sugar in the medium drops, only the high-affinity transporter remains active. The change in galactose concentration and that of the end metabolites was measured during the growth of MGGal^+^ in CDM-gal. The samples of the growth medium were taken during the first growth phase (OD_600_ of 0.12) and at the end of the stationary phase and analyzed by HPLC (Supplementary Figure 1). Although, MGGal^+^ performed mixed acid fermentation throughout the growth, more lactate was produced during the first growth phase than during the second one. Production of lactate as the main end product is usually associated with fast growth in *L. lactis*.

### Transcriptome Analyses

In order to identify genes involved in the growth of *L. lactis* MGGal^+^ in CDM-gal, we performed transcriptome studies using DNA-microarrays. Samples were taken along the growth of the strain in CDM supplemented with 5 mM galactose. In the absence of glucose, carbon catabolite repression (CCR) in *L. lactis* is relieved and many sugar metabolism-related genes are de-repressed. To exclude the effect of CCR from our transcriptome data, we compared *L. lactis* MGGal^+^ grown in the presence of galactose (5 mM) or fructose (5 mM). The results are presented in **Table 2**.

**Table 2:** Carbohydrate transport and utilization genes that are highly expressed in MGGal^+^ grown in CDM-gal as compared to CDM-fru – grown MGGal^+^.

Locus	Gene	Growth phase I	Growth phase II	Function
*llmg_2234*	*galT*	76.9	18.6	Galactose 1-P uridyltransferase
*llmg_2235*	*galK*	71.6	38.3	Galactokinase
*llmg_2236*	*galM*	55.8	28.9	Galactose mutarotase
*llmg_2237*	*galP*	44.2	24.2	Galactose permease
*llmg_2233*	*galE*	11.0	4.0	UDP-galactose-4-epimerase
*llmg_0438*	*ptcA*	57.9	50.7	Glucose/cellobiose PTS IIA component
*llmg_0439*	*llmg_0439*	33.1	17.1	LacI family transcriptional regulator
*llmg_0440*	*ptcC*	4.4	4.8	Glucose/cellobiose PTS IIC component
*llmg_0437*	*ptcB*	NA	NA	Glucose/cellobiose PTS IIB component
*llmg_0727*	*ptnD*	11.8	1.9	Glucose/mannose PTS IID component
*llmg_0728*	*ptnC*	3.3	2.1	Glucose/mannose PTS IIC component
*llmg_0729*	*ptnAB*	2.8	2.8	Glucose/mannose PTS IIAB component
*llmg_pseudo54*	*llmg_pseudo54*	5.8	5.0	Pseudogene IIC PTS component
*llmg_0739*	*malE*	16.5	13.1	Maltose ABC transporter substrate binding protein
*llmg_0738*	*malF*	11.0	9.6	Maltose ABC transporter
*llmg_0737*	*malG*	4.6	5.5	Maltose ABC transporter permease
*llmg_0746*	*malR*	2.1	2.3	Maltose operon transcriptional repressor
*llmg_0446*	*msmK*	22.3	16.1	Multiple sugar-binding transport ATP-binding protein
*llmg_1569*	*fruC*	–4.5	1.1	Tagatose-6-phosphate kinase
*llmg_1568*	*fruA*	–3.8	–1.3	PTS system, fructose specific IIBC components
*llmg_1570*	*fruR*	–3.1	1.3	Fructose operon transcriptional repressor

*Column “Growth phase I” shows fold upregulation of genes in cells harvested during early exponential phase (OD_600_ of 0.1); column “Growth phase II” – that of cells grown till late exponential phase (OD_600_ of 0.35). Only significantly expressed genes are included (Bayesian P < 0.001); “NA” specifies that the gene was not significantly expressed. The numbers between the two columns are not directly comparable.*

As expected, the fructose uptake- and metabolism genes *fruR, fruC, fruA* were fourfold up-regulated during growth in CDM-fru (Barriere et al., 2005), while the Leloir pathway enzymes were activated in cells cultured in galactose (the *gal* operon, on average 52-fold upregulation) (**Table 2**). Expression of the latter pathway is crucial for growth on galactose as MGGal^+^ does not possess the Tag-6-P route for galactose utilization. Next to the Leloir pathway encoding gene cluster, the glucose/cellobiose-specific PTS-specifying genes were strongly upregulated: the cytoplasmic IIA component-encoding *ptcA* – 58-fold, *llmg_0439*, which encodes a putative LacI-family transcription regulator and is present in the same operon, was up-regulated 43-fold. HTH-type transcriptional regulators similar to the one encoded by *llmg_0439* are annotated as phosphosugar-binding regulators, and can be found in many other bacteria (both *L. lactis* subspecies – *lactis* and *cremoris*; genus *Enterococcus, Lactobacillus, Clostridium, Bacillus, Cronobacter*). The location of *llmg_0439* in the *ptcBA* operon, adjacent to the *ptcC-celA* (which codes for an EIIC PTS component and a 6-P-β-glucosidase, respectively) cluster and its up-regulation suggests that the protein encoded by *llmg_0439* might be involved in the transcriptional regulation of the PTS PtcABC. The downstream gene *ptcC*, coding for the IIC membrane-integrated component of the same PTS, was up-regulated fourfold. The glucose/mannose-specific PTS-encoding operon *ptnABCD* was highly expressed during the growth phase I. Expression of *ptnD* and *ptnC* encoding the membrane-integrated components increased around 12- and 3-fold, respectively, while that of the cytoplasmic component *ptnAB* increased threefold. It has been previously suggested that galactose might be imported via a glucose transporter (Neves et al., 2010). Our transcriptome results appear to confirm this hypothesis.

Most of the sugar transporter genes with elevated transcription levels in the first growth phase were also upregulated during the later stage of growth, phase II (**Table 2**). In contrast to others, the gene of glucose/mannose – specific PTS component PtnD was around sixfold lower expressed during the second growth phase when compared to the first growth phase. The same was true, although to a lesser extent, for *ptnC*. GalP and the Leloir pathway enzymes were also expressed more moderately (two to fourfold lower upregulation) in the second phase of growth of MGGal^+^ in CDM-gal as compared to their expression levels during growth of MGGal^+^ in CDM-fru.

### *L. lactis* MG1363 Possesses More Than One PTS^Gal^

**FIGURE 2:**
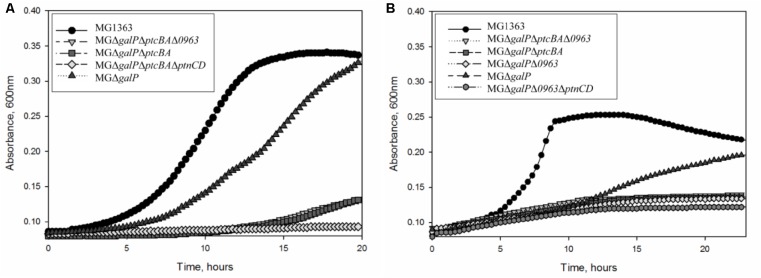
Growth of *L. lactis* strains in CDM: **(A)** supplemented with 50 mM galactose **(B)** supplemented with 5 mM galactose. Average growth of strains is shown.

To investigate whether the highly expressed glucose-specific PTSs play a role in galactose metabolism in *L. lactis* MG1363, we created a series of knock-out strains. First, we deleted the galactose permease gene *galP* from its chromosome. As expected, *L. lactis* MGΔ*galP* could still grow in CDM supplemented with galactose as the sole carbon source although the growth was slower than that of the wild-type strain (**Figure 2**). Next, to assess the role of PTS^Cel/Glu^ in galactose transport, *ptcBA* was deleted in MGΔ*galP*. This triple mutant was still able to grow in CDM-gal, but growth was seriously impaired. PtcBA are cytosolic EIIAB components that participate in the phosphorylation cascade and, thus, their role in galactose uptake might be indirect. To test whether PtcABC can really import galactose, the gene coding for the membrane-integrated component of the transporter, *ptcC*, was deleted from the chromosome of *L. lactis* MGΔ*galP*. The resulting strain *L. lactis* MGΔ*galP*Δ*ptcC* grew well in CDM-gal, suggesting that PtcC is not crucial for the uptake of this sugar. These results lead to two alternative explanations: either the role of PtcBA is not direct, but only regulatory, and galactose is imported by yet another transporter, or PtcAB are directly involved in transport of galactose, but they work together with a different EIIC component, not PtcC.

**FIGURE 3:**
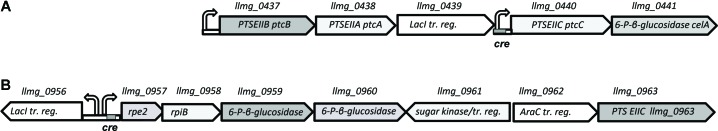
Genomic organization of PTS clusters on *L. lactis* MG1363 chromosome. **(A)** Genes coding for cellobiose/glucose-specific PTS PtcABC components **(B)** putative plant sugar utilization cluster, specifying a predicted PTS-EIIC component, Llmg_0963. Right/left bent arrows, promoter; *cre* – catabolite-responsive element, a CcpA operator sequence.

The genomic organization of the *ptc* cluster allows independent transcription of cytoplasmic and membrane-integrated components of the system (**Figure 3**). A separate promoter upstream of *ptcCcelA* contains a *cre* site and is under control of CcpA (Linares et al., 2010). The cytoplasmic components PtcAB have been shown to be promiscuous and to interact with two different PTS-EIIC subunits: PtcC for glucose and cellobiose transport and CelB for uptake of lactose and cellobiose (Aleksandrzak-Piekarczyk et al., 2011; Solopova et al., 2012).

Since it is not clear which PTS EIIC component is involved in galactose transport, we screened the genome sequence of *L. lactis* MG1363 for predicted EIIC components with unknown functions. The chromosome of *L. lactis* MG1363 harbors 12 gene clusters with various PTS elements. Three of these consist of a single EIIC component of unknown function. Llmg_pseudo_54 was confirmed to be a pseudogene by re-sequencing (Neves et al., 2010) leaving two possible candidates: *llmg_0963* and *llmg_1244*. The product of *llmg_1244* is a Lac-family protein homologous to cellobiose/lactose-specific PTS-EIIC components. The gene is located next to genes coding for cellulase and a transcriptional regulator of the Lac-I family. This cluster was recently shown to be involved in cellobiose uptake (Solopova et al., 2017). The second candidate, *llmg_0963* is present in a similar metabolic gene cluster. This gene codes for a putative PTS component with homology to cellobiose/lactose-specific PTS EIIC proteins of other Gram-positive bacteria (*Lactobacillus, Bacillus, Enterococcus*). The transcription of *llmg_0963* is regulated by LLMGnc_147, a small RNA which is highly expressed in media containing cellobiose or galactose. Cells in which LLMGnc_147 was overexpressed grew better in CDM-gal presumably because of higher expression of the *rpe2-llmg_0963* cluster (van der Meulen et al., 2016). In order to confirm that Llmg_0963 is indeed involved in galactose import the deletion mutant *L. lactis* MGΔ*galP*Δ*llmg_0963* was made and tested for its ability to grow in the presence of galactose. MGΔ*galP*Δ*llmg_0963* was still able to grow poorly in CDM-gal, but the growth of this strain was seriously affected. The combination of deletions of *galP, ptcBA*, and *llmg_0963* resulted in a similar phenotype with a prolonged lag-phase and low growth rate in CDM-gal, suggesting that galactose can still be taken up by these mutants (**Figure 2**). Slow growth of the cells indicated that residual galactose uptake might be a result of the activity of an unspecific sugar transporter.

**FIGURE 4:**
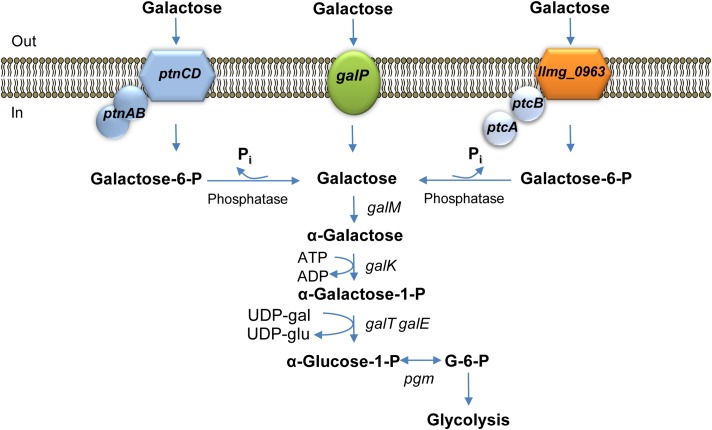
Galactose metabolism in *L. lactis* MG1363. *galP* encodes galactose permease; *llmg_0963ptcBA* and *ptnCDBA* code for low-affinity galactose PTSs. *galM*, galactose mutarotase; *galK*, galactokinase; *galT*, galactose 1-phosphate uridylyltransferase; *galE*, UDP-galactose 4-epimerase are the Leloir pathway enzymes-encoding genes; *pgm*,α-phosphoglucomutase.

Transcriptome analyses (**Table 2**) demonstrated that genes coding for PTS ^Man/Glu^ were highly expressed in *L. lactis* MGGal^+^ during early exponential growth in CDM-gal: *ptnD* was upregulated 12-fold, expression of *ptnC* and *ptnAB* increased three times. In order to assess a possible involvement of this glucose transport system in galactose import, *ptnCD* were deleted from the chromosomes of *L. lactis* MGΔ*galP*Δ*llmg_0963* and MGΔ*galP*Δ*llmg_0963*Δ*ptcBA*. When these combinations of genes were deleted the resulting strains *L. lactis* MGΔ*galP*Δ*llmg_0963*Δ*ptnCD* and MGΔ*galP*Δ*llmg_0963*Δ*ptcBA*Δ*ptnCD* did not grow on galactose as the sole carbon source for 20 h. **Figure 4** summarizes the results of this work and our proposed mechanism of galactose uptake and metabolism in *L. lactis* MG1363.

### The Genome of *L. lactis* MGGal^+^ Carries 29 Point-Mutations

In order to assess which genetic changes had occurred in MGGal^+^ and were responsible for the distinct growth pattern in CDM-gal, the whole genome of this strain was sequenced. Presumably, because of a mutation in the sequence of the DNA topoisomerase I gene, resulting in a premature STOP, MGGal^+^ became a mutator strain and acquired a total of 29 mutations (Supplementary Table 2). Two of these were present in the *gal* operon: a change from G to A in the promoter region (*-41*nt) and from C to T (R308C) in the coding region of *galP*. Although, the exact effect of these mutations on permease function is not clear, they apparently account for the biphasic growth of MGGal^+^ in CDM-gal. Presumably, the mutations decrease the expression and/or activity of the main galactose importer GalP, since MGGal^+^ exhibits a longer lag-phase and grows more slowly in CDM-gal than its parent strain.

## Discussion

Since the 1970s various laboratories have tried to elucidate galactose metabolism in *Lactococcus lactis*. Their studies often led to diverse outcomes, making it difficult to decide whether galactose uptake was PEP- or ATP-dependent in this organism and to determine the connection between galactose and lactose uptake and metabolism (Lee et al., 1973; LeBlanc et al., 1979; Thompson, 1980; Park and McKay, 1982; Crow et al., 1983; de Vos and Vaughan, 1994). It became clear that the transport of galactose via a PTS or a permease is usually coupled to a specific metabolic pathway and that the transport and utilization genes often are present in a gene cluster. PTS^Lac^-uptake is often coupled to the Tagatose-6-P pathway (the plasmid-encoded *lac* cluster), while transport via GalP leads to the Leloir pathway activation (the *gal* cluster) (Lee et al., 1973; LeBlanc et al., 1979; Grossiord et al., 1998). Later it was shown that some *Lactococcus* strains possess two galactose-specific transport systems – a permease and a PTS, even if they lack the lactose utilization plasmid, indicating that an alternative PTS^Gal^ exists (LeBlanc et al., 1979; Thompson, 1980; Neves et al., 2010). The identity of this transporter has thus far never been elucidated. Neves et al. (2010) hypothesized that a glucose transporter is able to additionally import galactose since high upregulation of *ptcA* was detected when the transcriptomes of glucose- and galactose-grown *L. lactis* NZ9000Δ*galP* were compared. PtcBA were proposed to interact with an unknown EIIC component to form a functional PTS (Neves et al., 2010). Increase in expression of the genes for subunits of two PTSs^Glu^, namely, *ptc*, and *ptn*, in galactose-grown cells led to the same conclusion in the work presented here. Using knock-out strains it was shown that the cytoplasmic PTS proteins PtcBA can form a functional transporter with an EIIC component encoded by *llmg_0963*. Llmg_0963 is the third membrane-integrated component with which PtcBA can presumably interact, besides the cellobiose/glucose-specific PtcC and the cellobiose/lactose-specific CelB. Only a few of the sequenced *Lactococcus* strains possess Llmg_0963. Besides MG1363 and its direct derivative NZ9000 an almost identical gene (99% identity) is present in the genomes of the *L. lactis* subsp. *cremoris* strains SK11 and A76. *L. lactis* SK11, A76, and NCDO712, the parent of MG1363, are dairy isolates used in starter cultures that often consist of a mixture of different strains (Bolotin et al., 2012). It was suggested that during such close contact of microorganisms horizontal gene transfer events could take place (Chamba and Jamet, 2008; Bolotin et al., 2012). However, if the protein encoded by *llmg_0963* is absent in other *L. lactis* strains, the identity of PTS^Gal^ in those strains still remains a mystery. It is likely that either PtnABCD performs this function alone, or PtcBA form a PTS^Gal^ with a different IIC component. In fact, the whole gene cluster containing *llmg_0963* is rare among lactococci and only present in the subspecies *cremoris*. It consists of pentose phosphate pathway genes coding for ribulose-P 3-epimerase (*rpe2*) and ribose-5-P isomerase B (*rpi*); downstream lay two 6-P-β-glucosidases (*llmg_0959* and *llmg_0960*), a sugar kinase and a putative transcriptional regulator (*llmg_0961*) homologous to xylose or N-acetyl-glucosamine kinase/regulator, and an AraC family (homologous to XylR) transcriptional regulator (*llmg_0962*). The EIIC-component-encoding *llmg_0963* is the last gene of the cluster. Overexpression of the small RNA LLMGnc_147 in response to cellobiose or galactose seems to increase expression of these genes (except for *llmg_0961*) (van der Meulen et al., 2016). The composition of the cluster and its induction by cellobiose is suggestive of an ancestral role in the uptake and metabolism of plant sugars. It has previously been proposed that during adaptation to milk *L. lactis* has lost many of the plant-niche-specific genes (Godon et al., 1993; Siezen et al., 2005; Bachmann et al., 2012). Others became silenced and/or have adopted other functions, such as the cellobiose transporter CelB, the gene of which could acquire a functional promoter due to a high-frequency mutation upon growth of *L. lactis* MG1363 in lactose-containing medium. Thus, the mutant strain could transport lactose and grow (Solopova et al., 2012). The 102-nt-long small RNA LLMGnc_147, which presumably stabilizes the transcript of the *rpe2-llmg_0963*, is highly conserved among lactococci of the subspecies *cremoris*; most strains belonging to subsp. *lactis* possess a homologous sRNA with 93% identity to that of LLMGnc_147. Since these strains do not contain a cluster homologous to *rpe2*-*llmg_0963*, the function of the sRNA in these subsp. *lactis* strains is enigmatic. The fact that we did not detect overexpression of the *rpe2*-*llmg_0963* gene cluster in the DNA microarray analyses in this and the previous study (Neves et al., 2010) might be explained by low expression levels of the cluster genes under conditions tested. Weak expression of the cluster would hamper the separation of the signal from the background noise. The fact that deletion of *llmg_0963* did not completely abolish the Gal^+^ phenotype of *L. lactis* MGΔ*galP* is in agreement with the low expression of the gene. Apparently, galactose alone does not serve as a signal for the induction of the *rpe2*-*llmg_0963* gene cluster. The same is true for LLMGnc_147 (van der Meulen et al., 2016). Cellobiose was shown to be the strongest inducer of the expression of the latter (van der Meulen et al., 2016).

PTS^Man/Glu^ PtnABCD is the second PTS involved in galactose transport in *L. lactis* MG1363. Similarly, glucose/mannose PTS^Man^ of *S. mutans* and *S. gordonii* play important roles in galactose metabolism: the transporter imports galactose, while its cytoplasmic components EIIAB regulate the expression of multiple PTSs next to CcpA-mediated CCR (Zeng et al., 2010; Tong et al., 2011). To minimize possible indirect regulation effects and to solely evaluate the transport possibilities of PtnABCD, we here deleted only the IICD-components-encoding genes *ptnCD* from the chromosome of *L. lactis* MG1363. In some cases, the membrane-spanning components of PTSs are involved in facilitated diffusion and allow transport of galactose or fructose in *E. coli* (via the glucose-specific PTS) (Kornberg and Riordan, 1976; Kornberg et al., 2000). Mannose PTS was shown to facilitate uptake of galactose and trehalose in *S. typhimurium* (Postma, 1976; Postma et al., 1986) and of xylose in *L. pentosus, L. plantarum*, and *L. casei* (Chaillou et al., 1999).

Since PTS systems are involved in regulation and sensing of the sugar availability, it can be difficult to untangle whether the mutant phenotype is due to the direct involvement of the deleted transport system component, or it is an indirect effect of the disrupted regulatory cascade. Additionally, compensating mutations elsewhere on the chromosome might occur and only the resequencing of the whole genome of the strain can pinpoint such mutation. Various cases demonstrated how an unidentified gene or even a pseudogene exposes its activity when a strong selection pressure is applied (Vaughan et al., 2001; Bongers et al., 2003; Gaspar et al., 2007; Aleksandrzak-Piekarczyk et al., 2011; Solopova et al., 2012, 2017), indicating that caution should be taken in the interpretation of knock-out results.

The chemically-mutagenized Gal^+^ isolate *L. lactis* MGGal^+^ demonstrated a biphasic growth in CDM-gal. Genome re-sequencing revealed 29 mutations in the chromosome of this strain making it difficult to pinpoint the one(s) responsible for its specific behavior in CDM-gal. Among the mutations are two in the *galP* region (in the promoter and in the coding sequence). While the mutation in the promoter region seems to be neutral, a change from Arg 308 to Cys in the conserved region of GalP (462 aa in total) could have a drastic effect on protein function. The lower growth rate of MGGal^+^ in CDM-gal is in agreement with this hypothesis. The higher biomass yield of *L. lactis* MGGal^+^ on galactose could be the result of a lower acidification rate of the medium. However, the high number of mutations in the genome of this strain makes it almost impossible to associate its phenotype with the effect of a specific mutation.

## Author Contributions

AS and HB designed and carried out the experiments, analyzed the data, and wrote the manuscript. BT analyzed the data and wrote the manuscript. JK designed the experiments, analyzed the data, and wrote the manuscript. OK conceived and designed the experiments, analyzed the data, and wrote the manuscript.

## Conflict of Interest Statement

The authors declare that the research was conducted in the absence of any commercial or financial relationships that could be construed as a potential conflict of interest.
