# Supplementary material for: Further Elucidation of Galactose Utilization in Lactococcus lactis MG1363
Source: Front Microbiol. 2018 Aug 3;9:1803. doi: 10.3389/fmicb.2018.01803 (PMC6085457; doi:10.3389/fmicb.2018.01803)
Supplement: Supplementary file 1 [file Table_1.DOCX]

Supplementary Material

Further elucidation of galactose utilization in *Lactococcus lactis* MG1363

Ana Solopova, Herwig Bachmann, Bas Teusink, Jan Kok and Oscar P. Kuipers^*^

*** Correspondence:** Oscar P. Kuipers: o.p.kuipers@rug.nl

**Supplementary Table 1.** Primers used in this study. Restriction sites are underlined.

| Primer | Sequence 5’🡪3’ |
| --- | --- |
| KogalPF1XbaI | GCATTCTAGATTATGAAGCGATTTCATAGC |
| KogalPF3BamHI | GCATGGATCCTGAAGAACGTTTGAAATAAG |
| KogalPRev2BamHI | GCATGGATCCATGGGAATCCTCCTTTATAC |
| KogalPRev4XhoI | CGTACTCGAGGCGCTTGTTCTTTCTCAAGG |
| KoPtcBA1FXbaI | GCATTCTAGATCGGGATTTGGCAAGTCAGG |
| KoPtcBA2RevBamHI | GCATGGATCCGCACATGCAAGTGCAATAAC |
| KoPtcBA3FBamHI | GCATGGATCCCAAGAACGACGCCTTCAAGC |
| KoPtcBA4RevXhoI | CGTACTCGAGGGTAATTGCCTCGTTAAGTC |
| Ko0963F3BamHI | TGCATGGATCCGAATTAGAGGGTTCAGAAAC |
| Ko0963Fw1XbaI | AAAATTCTAGAAATTATGGTATGCATTATAG |
| Ko0963Rev2BamHI | TGCATGGATCCTCTTTAGATTCACTCCTTTAAC |
| Ko0963Rev4KpnI | TTTTAGGTACCAGACTTCTACTGACAGATTC |
| KoptnCDF1XbaI | GCATTCTAGAGTGAATTCGCTGCAGGCATC |
| KoptnCDRev2BamHI | GCATGGATCCCAGATAAAACACCGTATTCC |
| KoptnCDF3BamHI | GCATGGATCCTGGTCTTTAGATTATTAATC |
| KoptnCDRev4XhoI | CGTACTCGAGAATGCTATGGCAAGCAATGG |
| KoPtcCF1XbaI | GCATTCTAGAGTAATTGCTTTATCCATTTC |
| KoPtcCRev2BamHI | GCATGGATCCGGTTGAACAGTCTCCTTTAC |
| KoPtcCF3BamHI | GCATGGATCCTTAATGCTGCCGAATAATTG |
| KoPtcCRev4XhoI | CGTACTCGAGGCCGCTTGATACATGATTTC |
