# Supplementary material for: Further Elucidation of Galactose Utilization in Lactococcus lactis MG1363
Source: Front Microbiol. 2018 Aug 3;9:1803. doi: 10.3389/fmicb.2018.01803 (PMC6085457; doi:10.3389/fmicb.2018.01803)
Supplement: Supplementary file 2 [file Table_2.DOCX]

Supplementary Material

Further elucidation of galactose utilization in *Lactococcus lactis* MG1363

Ana Solopova, Herwig Bachmann, Bas Teusink, Jan Kok and Oscar P. Kuipers^*^

*** Correspondence:** Oscar P. Kuipers: o.p.kuipers@rug.nl

**Supplementary Table 2.** Mutations in the genome of *L. lactis* MGGal^+^ as determined by whole-genome re-sequencing.

| Position | Mutation | Annotation | Gene/locus | Predicted function |
| --- | --- | --- | --- | --- |
| 142,07 | A→G | E228G (GAA→GGA) | *llmg_0146* | aryl‑alcohol dehydrogenase |
| 1,092,096 | Δ1 bp | coding (2776/4050 nt) | *llmg_1127* | cell wall surface anchor family protein |
| 1,135,131 | C→T | R451C (CGC→TGC) | *aguA* | AguA protein |
| 1,149,866 | G→A | G137E (GGG→GAG) | *ilvE* | branched‑chain amino acid aminotransferase |
| 1,158,763 | C→T | C13C (TGC→TGT) | *llmg_1186* | hypothetical protein |
| 1,192,659 | G→A | D361N (GAT→AAT) | *mgtA* | cation‑transporting P‑ATPase |
| 1,205,061 | G→A | intergenic (‑282/+359) | *llmg_1234*/ *llmg_1235* | hypothetical protein/cyclic nucleotide‑binding domain‑containing protein |
| 1,205,261 | G→A | intergenic (‑482/+159) | *llmg_1234*/ *llmg_1235* | hypothetical protein/cyclic nucleotide‑binding domain‑containing protein |
| 1,223,555 | Δ1 bp | intergenic (+243/+302) | *llmg_1251*/ *llmg_1252* | hypothetical protein/hypothetical protein |
| 1,223,557 | 2 bp→AG | intergenic (+245/+299) | *llmg_1251*/ *llmg_1252* | hypothetical protein/hypothetical protein |
| 1,236,087 | G→A | intergenic (+1218/‑318) | *llmg_1267*/ *xerS* | hypothetical protein/site‑specific tyrosine recombinase XerS |
| 1,238,096 | G→A | intergenic (+265/‑218) | *llmg_1269*/ *xerS* | hypothetical protein/site‑specific tyrosine recombinase XerS |
| 1,242,451 | C→T | W176* (TGG→TGA) | *topA* | DNA topoisomerase I |
| 1,364,819 | G→A | R691* (CGA→TGA) | *llmg_1391* | hypothetical protein |
| 1,416,325 | C→T | S228N (AGC→AAC) | *llmg_1448* | hypothetical protein |
| 1,466,764 | G→A | R58C (CGT→TGT) | *llmg_1495* | hypothetical protein |
| 1,598,251 | C→T | G248R (GGA→AGA) | *tagG* | teichoic acid ABC transporter permease |
| 1,778,942 | C→T | G164D (GGT→GAT) | *srtC* | sortase |
| 1,800,889 | G→A | G231E (GGA→GAA) | *thrA* | aspartate kinase |
| 1,927,919 | C→T | G354S (GGT→AGT) | *atpD* | ATP synthase F0F1 subunit beta |
| 2,019,825 | G→A | R264C (CGT→TGT) | *gidA* | tRNA uridine 5‑carboxymethylaminomethyl modification protein GidA |
| 2,046,450 | G→A | T96I (ACA→ATA) | *trmH* | tRNA/rRNA methyltransferase |
| 2,179,399 | C→T | A279T (GCC→ACC) | *aspS* | aspartyl‑tRNA synthetase |
| 2,200,043 | G→A | R308C (CGC→TGC) | *galP* | galactose permease |
| 2,201,005 | G→A | intergenic (‑41/+266) | *galP*/ *llmg_2238* | galactose permease/hypothetical protein |
| 2,202,323 | G→A | intergenic (‑300/+908) | *llmg_2238*/ *nadR* | hypothetical protein/nicotinamide‑nucleotide adenylyltransferase |
| 2,218,982 | C→T | V190I (GTC→ATC) | *ps508* | phage protein |
| 2,223,096 | G→A | E80K (GAA→AAA) | *ps504* | hypothetical protein |
| 2,236,473 | G→A | Q64* (CAA→TAA) | *bcrA* | bacitracin transport ATP‑binding protein |
