# Supplementary material for: Further Elucidation of Galactose Utilization in Lactococcus lactis MG1363
Source: Front Microbiol. 2018 Aug 3;9:1803. doi: 10.3389/fmicb.2018.01803 (PMC6085457; doi:10.3389/fmicb.2018.01803)
Supplement: Supplementary file 3 [file Data_Sheet_1.docx]

Supplementary Material

Further elucidation of galactose utilization in *Lactococcus lactis* MG1363

Ana Solopova, Herwig Bachmann, Bas Teusink, Jan Kok and Oscar P. Kuipers^*^

*** Correspondence:** Oscar P. Kuipers: o.p.kuipers@rug.nl

**Supplementary Figure 1.** Galactose and organic acid concentrations of culture supernatant (CDM-gal) of *L. lactis* MGGal^+^. Light grey, samples taken during growth phase I (OD_600_ of 0.12); dark grey, samples taken from stationary phase (after growth phase II). Note that most lactate is produced during the first growth stage when cells are growing fast: lactate/acetate fraction during the Growth phase I is ~0.3; after Growth phase II it is ~0.096. Error bars represent standard deviation (n=2).
